# Supplementary material for: Volatile anesthetics versus total intravenous anesthesia in patients undergoing coronary artery bypass grafting: An updated meta-analysis and trial sequential analysis of randomized controlled trials
Source: PLoS One. 2019 Oct 29;14(10):e0224562. doi: 10.1371/journal.pone.0224562 (PMC6818786; doi:10.1371/journal.pone.0224562)
Supplement: S1 Table — (DOCX) [file pone.0224562.s001.docx]

**S1 Table. Search strategy in PubMed**

PubMed 06.19.2019

| #1 | (Anesthetics, Inhalation[MeSH Terms]) OR (((((((Inhal*[Title/Abstract]) OR gas[Title/Abstract]) OR gases[Title/Abstract]) OR respiration[Title/Abstract]) OR volatile[Title/Abstract])) AND anesthetic*[Title/Abstract]) | 19955 |
| --- | --- | --- |
| #2 | (((((Halothane[MeSH Terms]) OR Halothane[Title/Abstract]) OR fluothane[Title/Abstract]) OR bromoclorotrifluoroethane[Title/Abstract]) OR Ftorotan[Title/Abstract]) OR Narcotan[Title/Abstract] | 19659 |
| #3 | ((((Sevoflurane[MeSH Terms]) OR Sevoflurane[Title/Abstract]) OR Sevorane[Title/Abstract]) OR Ultane[Title/Abstract]) OR Sevo[Title/Abstract] | 9045 |
| #4 | (((desflurane[MeSH Terms]) OR desflurane[Title/Abstract]) OR suprane[Title/Abstract]) OR tetrafluoroethane[Title/Abstract] | 2439 |
| #5 | (((isoflurane[MeSH Terms]) OR isoflurane[Title/Abstract]) OR Forane[Title/Abstract]) OR IsoFlu[Title/Abstract] | 14018 |
| #6 | ((((((enflurane[MeSH Terms]) OR enflurane[Title/Abstract]) OR Etran[Title/Abstract]) OR Enlirane[Title/Abstract]) OR Ethrane[Title/Abstract]) OR Alyrane[Title/Abstract]) OR Enfran[Title/Abstract] | 3716 |
| #7 | (((((((methoxyflurane[MeSH Terms]) OR methoxyflurane[Title/Abstract]) OR Methofluranum[Title/Abstract]) OR Penthrane[Title/Abstract]) OR Pentrane[Title/Abstract]) OR Anecotan[Title/Abstract]) OR metoksyfluran[Title/Abstract]) OR metoxiflurano[Title/Abstract] | 2346 |
| #8 | ((Ether[MeSH Terms]) OR Ether*[Title/Abstract]) OR ethyl oxide[Title/Abstract] | 60645 |
| #9 | #1 OR #2 OR #3 OR #4 OR #5 OR #6 OR #7 OR #8 | 107405 |
| #10 | ((((Coronary Artery Bypass[MeSH Terms]) OR ((Coronary[Title/Abstract]) AND Artery[Title/Abstract])) OR Aortocoronary[Title/Abstract]) OR CABG[Title/Abstract]) OR OPCAB[Title/Abstract] | 211602 |
| #11 | ((clinical[Title/Abstract] AND trial[Title/Abstract]) OR clinical trials as topic[MeSH Terms] OR clinical trial[Publication Type] OR random*[Title/Abstract] OR random allocation[MeSH Terms] OR therapeutic use[MeSH Subheading]) | 5288765 |
| #12 | #9 AND #10 AND #11 | 685 |
