# Supplementary material for: Volatile anesthetics versus total intravenous anesthesia in patients undergoing coronary artery bypass grafting: An updated meta-analysis and trial sequential analysis of randomized controlled trials
Source: PLoS One. 2019 Oct 29;14(10):e0224562. doi: 10.1371/journal.pone.0224562 (PMC6818786; doi:10.1371/journal.pone.0224562)
Supplement: S3 Table — (DOCX) [file pone.0224562.s003.docx]

**S3 Table. Reference list of included RCTs**

| **Author** | **Reference** |
| --- | --- |
| 1. Amr 2010 | Amr YM, Yassin IM. Cardiac protection during on-pump coronary artery bypass grafting: ischemic versus isoflurane preconditioning. Seminars in cardiothoracic and vascular anesthesia. 2010;14(3):205-211. |
| 2. Azab 2000 | El Azab SR, Scheffer GJ, Rosseel PM, De Lange JJ. Induction and maintenance of anaesthesia with sevoflurane in comparison to high dose opioid during coronary artery bypass surgery. European journal of anaesthesiology. 2000;17(5):336-338. |
| 3. Azab 2003 | El Azab SR, Rosseel PMJ, De Lange JJ, et al. Effect of sevoflurane on the ex vivo secretion of TNF-alpha during and after coronary artery bypass surgery. European journal of anaesthesiology. 2003;20(5):380-384. |
| 4. Baki 2013 | Baki ED, Aldemir M, Kokulu S, Koca HB, Ela Y, Sivaci RG, et al. Comparison of the effects of desflurane and propofol anesthesia on the inflammatory response and S100beta protein during coronary artery bypass grafting. Inflammation. 2013;36(6):1327-1333. |
| 5. Ballester 2011 | Ballester M, Llorens J, Garcia-de-la-Asuncion J, et al. Myocardial oxidative stress protection by sevoflurane vs. propofol: a randomised controlled study in patients undergoing off-pump coronary artery bypass graft surgery. European journal of anaesthesiology. 2011;28(12):874-881. |
| 6. Bein 2005 | Bein B, Renner J, Caliebe D, et al. Sevoflurane but not propofol preserves myocardial function during minimally invasive direct coronary artery bypass surgery. Anesthesia and analgesia. 2005;100(3):610-616. |
| 7. Bein 2008 | Bein B, Renner J, Caliebe D, Hanss R, Bauer M, Fraund S, et al. The effects of interrupted or continuous administration of sevoflurane on preconditioning before cardio-pulmonary bypass in coronary artery surgery: Comparison with continuous propofol. Anaesthesia. 2008;63(10):1046-1055. |
| 8. Belhomme 1999 | Belhomme D, Peynet J, Louzy M, Launay JM, Kitakaze M, Menasche P. Evidence for preconditioning by isoflurane in coronary artery bypass graft surgery. Circulation. 1999;100(19 Suppl):II340-344. |
| 9. Bharti 2008 | Bharti N, Chari P, Thingnam SS, Arora S. Comparison of Haemodynamic and Cardiovascular Effects of VIMA with Sevoflurane Versus TIVA with Propofol in Patients Undergoing Coronary Artery Bypass Surgery. Indian journal of anaesthesia. 2008;52(6):805-812. |
| 10. Bi 2008 | Bi Y, Jiang M, Sun L, Wang M, Wang B. Effects of propofol on cerebral oxygen metabolism and psychoneurotic function in patients undergoing coronary artery bypass grafting with extracorporeal circulation. Medical journal of Qilu. 2008;23(1):30-32. |
| 11. Botero 2000 | Botero CA, Smith CE, Holbrook C, et al. Total intravenous anesthesia with a propofol-ketamine combination during coronary artery surgery. Journal of cardiothoracic and vascular anesthesia. 2000;14(4):409-415. |
| 12. Chang 2007 | Chang FL, Lin SL, Tsai CS, Yeh CC, Wu CT, Wong CS. Closed-circuit isoflurane-based anesthesia provides better fast-tracking anesthesia than fentanyl/propofol-based anesthesia for off-pump coronary artery bypass graft surgery. Acta anaesthesiologica Taiwanica. 2007;45(3):135-139. |
| 13. Chao 2010 | Chao C, Li L, Lei W, Fei J. Effects of isoflurane and propofol on cognitive function after off-pump coronary artery bypass grafting. Chinese journal of behavioral medicine and brain science. 2010;19(11):970-971. |
| 14. Chen 2014 | Chen F, Miao J, Lin L. Effect of sevoflurane on early pneumonia response after extracorporeal circulation. Journal of Wenzhou Medical University. 2014;44(8):570-573. |
| 15. Conzen 2003 | Conzen PF, Fischer S, Detter C, Peter K. Sevoflurane provides greater protection of the myocardium than propofol in patients undergoing off-pump coronary artery bypass surgery. Anesthesiology. 2003;99(4):826-833. |
| 16. De Hert 2003 | [De Hert SG](https://www.ncbi.nlm.nih.gov/pubmed/?term=De%20Hert%20SG%5BAuthor%5D&cauthor=true&cauthor_uid=12883404), [Cromheecke S](https://www.ncbi.nlm.nih.gov/pubmed/?term=Cromheecke%20S%5BAuthor%5D&cauthor=true&cauthor_uid=12883404), [ten Broecke PW](https://www.ncbi.nlm.nih.gov/pubmed/?term=ten%20Broecke%20PW%5BAuthor%5D&cauthor=true&cauthor_uid=12883404), [Mertens E](https://www.ncbi.nlm.nih.gov/pubmed/?term=Mertens%20E%5BAuthor%5D&cauthor=true&cauthor_uid=12883404), [De Blier IG](https://www.ncbi.nlm.nih.gov/pubmed/?term=De%20Blier%20IG%5BAuthor%5D&cauthor=true&cauthor_uid=12883404), [Stockman BA](https://www.ncbi.nlm.nih.gov/pubmed/?term=Stockman%20BA%5BAuthor%5D&cauthor=true&cauthor_uid=12883404), et al. Effects of propofol, desflurane, and sevoflurane on recovery of myocardial function after coronary surgery in elderly high-risk patients. [Anesthesiology.](https://www.ncbi.nlm.nih.gov/pubmed/?term=Effects+of+Propofol%2C+Desflurane%2C+and+Sevoflurane+on+Recovery+of+Myocardial+Function+after+Coronary+Surgery+in+Elderly+High-risk+Patients) 2003;99(2):314-323. |
| 17. De Hert 2004a | De Hert SG, Van der Linden PJ, Cromheecke S, et al. Cardioprotective properties of sevoflurane in patients undergoing coronary surgery with cardiopulmonary bypass are related to the modalities of its administration. Anesthesiology. 2004;101(2):299-310. |
| 18. De Hert 2004b | [De Hert SG](https://www.ncbi.nlm.nih.gov/pubmed/?term=De%20Hert%20SG%5BAuthor%5D&cauthor=true&cauthor_uid=15277911), [Van der Linden PJ](https://www.ncbi.nlm.nih.gov/pubmed/?term=Van%20der%20Linden%20PJ%5BAuthor%5D&cauthor=true&cauthor_uid=15277911), [Cromheecke S](https://www.ncbi.nlm.nih.gov/pubmed/?term=Cromheecke%20S%5BAuthor%5D&cauthor=true&cauthor_uid=15277911), [Meeus R](https://www.ncbi.nlm.nih.gov/pubmed/?term=Meeus%20R%5BAuthor%5D&cauthor=true&cauthor_uid=15277911), [Nelis A](https://www.ncbi.nlm.nih.gov/pubmed/?term=Nelis%20A%5BAuthor%5D&cauthor=true&cauthor_uid=15277911), [Van Reeth V](https://www.ncbi.nlm.nih.gov/pubmed/?term=Van%20Reeth%20V%5BAuthor%5D&cauthor=true&cauthor_uid=15277911), et al. Cardioprotective Properties of Sevoflurane in Patients Undergoing Coronary Surgery with Cardiopulmonary Bypass Are Related to the Modalities of Its Administration. [Anesthesiology.](https://www.ncbi.nlm.nih.gov/pubmed/15277911) 2004;101(2):299-310. |
| 19. De Hert 2009 | De Hert S, Vlasselaers D, Barbe R, et al. A comparison of volatile and non volatile agents for cardioprotection during on-pump coronary surgery. Anaesthesia. 2009;64(9):953-960 |
| 20. Drenger 2008 | Drenger B, Gilon D, Chevion M, Elami A, Meroz Y, Milgalter E, et al. Myocardial metabolism altered by ischemic preconditioning and enflurane in off-pump coronary artery surgery. Journal of cardiothoracic and vascular anesthesia. 2008;22(3):369-376. |
| 21. Driessen 1997 | Driessen JJ, Giart M. Comparison of isoflurane and midazolam as hypnotic supplementation to moderately high-dose fentanyl during coronary artery bypass grafting: effects on systemic hemodynamics and early postoperative recovery profile. Journal of cardiothoracic and vascular anesthesia. 1997;11(6):740-745. |
| 22. Flier 2010 | Flier S, Post J. Concepcion AN, Kappen TH, Kalkman CJ, Buhre WF. Influence of propofol-opioid vs isoflurane-opioid anaesthesia on postoperative troponin release in patients undergoing coronary artery bypass grafting. British journal of anaesthesia. 2010;105(2):122-130. |
| 23. Frassdorf 2009 | Frassdorf J, Borowski A, Ebel D, Feindt P, Hermes M, Meemann T, et al. Impact of preconditioning protocol on anesthetic-induced cardioprotection in patients having coronary artery bypass surgery. Journal of Thoracic and Cardiovascular Surgery. 2009 June;137(6):1436-42.e2. |
| 24. Garcia 2005 | Garcia C, Julier K. Bestmann L, et al. Preconditioning with sevoflurane decreases PECAM-1 expression and improves one-year cardiovascular outcome in coronary artery bypass graft surgery. British journal of anaesthesia. 2005;94(2):159-165. |
| 25. Gravel 1999 | Gravel NR, Searle NR, Taillefer J, Carrier M, Roy M, Gagnon L. Comparison of the hemodynamic effects of sevoflurane anesthesia induction and maintenance vs TIVA in CABG surgery. Journal canadien d'anesthesie. Canadian journal of anaesthesia. 1999;46(3):240-246. |
| 26. Guarracino 2006 | Guarracino F, Landoni G, Tritapepe L, et al. Myocardial damage prevented by volatile anesthetics: a multicenter randomized controlled study. Journal of cardiothoracic and vascular anesthesia. 2006;20(4):477-483. |
| 27. Hall 1991 | Hall RI, Murphy JT, Moffitt EA, Landymore R, Pollak PT, Poole L. A comparison of the myocardial metabolic and haemodynamic changes produced by propofol-sufentanil and enflurane-sufentanil anaesthesia for patients having coronary artery bypass graft surgery. Journal canadien d'anesthesie. Canadian journal of anaesthesia. 1991;38(8):996-1004. |
| 28. Hall 1993 | Hall RI, Murphy JT, Landymore R, Pollak PT, Doak G, Murray M. Myocardial metabolic and hemodynamic changes during propofol anesthesia for cardiac surgery in patients with reduced ventricular function. Anesthesia and analgesia. 1993;77(4):680-689. |
| 29. Haroun-Bizri 2001 | Haroun-Bizri S, Khoury SS, Chehab IR, Kassas CM, Baraka A. Does isoflurane optimize myocardial protection during cardiopulmonary bypass? Journal of cardiothoracic and vascular anesthesia. 2001;15(4):418-421. |
| 30. Helman 1992 | Helman JD, Leung JM, Bellows WH, Pineda N, Roach GW, Reeves IJD, et al. The risk of myocardial ischemia in patients receiving desflurane versus sufentanil anesthesia for coronary artery bypass graft surgery. Anesthesiology. 1992;77(1):47-62. |
| 31. Hofland 2017 | Hofland J, Ouattara A, Fellahi JL, Gruenewald M, Hazebroucq J, Ecoffey C, et al. Effect of Xenon Anesthesia Compared to Sevoflurane and Total Intravenous Anesthesia for Coronary Artery Bypass Graft Surgery on Postoperative Cardiac Troponin Release: an International, Multicenter, Phase 3, Single-blinded, Randomized Noninferiority Trial. Anesthesiology. 2017;127(6):918-933. |
| 32. Huang 2011 | Huang Z, Zhong X, Irwin MG, Ji S, Wong GT, Liu Y, et al. Synergy of isoflurane preconditioning and propofol postconditioning reduces myocardial reperfusion injury in patients. Clinical Science. 2011;121(2):57-69. |
| 33. Jerath 2015 | Jerath A, Beattie SW, Chandy T, Karski J, Djaiani G, Rao V, et al. Volatile-based short-term sedation in cardiac surgical patients: A prospective randomized controlled trial. Critical care medicine. 2015;43(5):1062-1069. |
| 34. Ji 2013 | Ji S, Liu B. The protective effect of sevoflurane on myocardium during off-pump coronary artery bypass grafting. Medical & pharmaceutical journal of Chinese people’s liberation army. 2013;25(3):64-68. |
| 35. Jia 2015 | Jia L, Dong R, Zhang F, Wang W, Lu H, Luo Y, et al. Propofol Provides More Effective Protection for Circulating Lymphocytes Than Sevoflurane in Patients Undergoing Off-Pump Coronary Artery Bypass Graft Surgery. Journal of cardiothoracic and vascular anesthesia. 2015;29(5):1172-1179. |
| 36. Jia 2018 | Jia Y, Han Z. Effects of different anesthesia methods on cognitive function after coronary artery bypass grafting in elderly patients with coronary heart disease. China Practical Medical. 2018;13(10):110-111. |
| 37. Julier 2003 | Julier K, Da Silva R, Garcia C, Bestmann L, Frascarolo P, Zollinger A, et al. Preconditioning by sevoflurane decreases biochemical markers for myocardial and renal dysfunction in coronary artery bypass graft surgery: A double-blinded, placebo-controlled, multicenter study. Anesthesiology. 2003;98(6):1315-1327. |
| 38. Kendall 2004 | Kendall JB, Russell GN, Scawn ND, Akrofi M, Cowan CM, Fox MA. A prospective, randomised, single-blind pilot study to determine the effect of anaesthetic technique on troponin T release after off-pump coronary artery surgery. Anaesthesia. 2004;59(6):545-549. |
| 39. Kim 2011 | Kim TY, Kim DK, Yoon TG, et al. Myocardial injury in remifentanil-based anaesthesia for off-pump coronary artery bypass surgery: an equipotent dose of sevoflurane versus propofol. Anaesthesia and intensive care. 2011;39(3):418-425. |
| 40. Landoni 2019 | Landoni G, Lomivorotov VV, Nigro Neto C, Monaco F, Pasyuga VV, Bradic N, et al. Volatile Anesthetics versus Total Intravenous Anesthesia for Cardiac Surgery. The New England journal of medicine. 2019;380(13):1214-1225. |
| 41. Law-Koune 2006 | Law-Koune JD, Raynaud C, Liu N, Dubois C, Romano M, Fischler M. Sevoflurane-remifentanil versus propofol-remifentanil anesthesia at a similar bispectral level for off-pump coronary artery surgery: no evidence of reduced myocardial ischemia. Journal of cardiothoracic and vascular anesthesia. 2006;20(4):484-492. |
| 42. Lee 2006 | Lee MC, Chen CH, Kuo MC, Kang PL, Lo A, Liu K. Isoflurane preconditioning-induced cardio-protection in patients undergoing coronary artery bypass grafting. European journal of anaesthesiology. 2006;23(10):841-7. |
| 43. Lemoine 2018 | Lemoine S, Zhu L, Gerard JL, Hanouz JL. Sevoflurane-induced cardioprotection in coronary artery bypass graft surgery: randomised trial with clinical and ex-vivo endpoints. Anaesthesia, critical care & pain medicine. 2018;37(3):217-223. |
| 44. Leung 1991 | Leung JM, Goehner P, O'Kelly BF, et al. Isoflurane anesthesia and myocardial ischemia: comparative risk versus sufentanil anesthesia in patients undergoing coronary artery bypass graft surgery. The SPI (Study of Perioperative Ischemia) Research Group. Anesthesiology. 1991;74(5):838-847. |
| 45. Li 2010 | Li L, Lei W, Fei J, Chao C. Effects of different anesthesia methods on cognitive function after coronary artery bypass grafting in elderly patients with coronary heart disease. Chinese journal of geriatric heart brain and vessel diseases. 2010;12(11):1002-1004. |
| 46. Likhvantsev 2016 | Likhvantsev VV, Landoni G, Levikov DI, Grebenchikov OA, Skripkin YV, Cherpakov RA. Sevoflurane Versus Total Intravenous Anesthesia for Isolated Coronary Artery Bypass Surgery With Cardiopulmonary Bypass: A Randomized Trial. Journal of cardiothoracic and vascular anesthesia. 2016;30(5):1221-1227. |
| 47. Liu 2012 | Liu X. Clinical study on myocardial protection during OPCABG surgery with sevoflurane total inhalation anesthesia [M]: central south university; 2012. |
| 48. Lorsomradee 2006 | Lorsomradee S, Cromheecke S, Lorsomradee S, De Hert SG. Effects of sevoflurane on biomechanical markers of hepatic and renal dysfunction after coronary artery surgery. Journal of cardiothoracic and vascular anesthesia. 2006;20(5):684-690. |
| 49. Lu 2003 | Lu CC, Ho ST, Wang JJ, et al. Minimal low-flow isoflurane-based anesthesia benefits patients undergoing coronary revascularization via preventing hyperglycemia and maintaining metabolic homeostasis. Acta anaesthesiologica Sinica. 2003;41(4):165-172. |
| 50. Luo 2016 | Luo Y, Yue Y. Effects of sevoflurane and propofol anesthesia on postoperative outcomes of patients undergoing off-pump coronary artery bypass grafting. Beijing Medical Journal. 2016;38(1):35-38. |
| 51. Meco 2007 | Meco M, Cirri S, Gallazzi C, Magnani G, Cosseta D. Desflurane preconditioning in coronary artery bypass graft surgery: a double-blinded, randomised and placebo-controlled study. European Journal of Cardio-thoracic Surgery. 2007;32(2):319-325. |
| 52. Mora 1995 | Mora CT, Dudek C, Torjman MC, White PF. The effects of anesthetic technique on the hemodynamic response and recovery profile in coronary revascularization patients. Anesthesia and analgesia. 1995;81(5):900-910. |
| 53. Mrozinski 2014 | Mrozinski P, Lango R, Biedrzycka A, Kowalik MM, Pawlaczyk R, Rogowski J. Comparison of haemodynamics and myocardial injury markers under desflurane vs. propofol anaesthesia for off-pump coronary surgery. A prospective randomised trial. Anaesthesiology intensive therapy. 2014;46(1):4-13. |
| 54. Myles 1997 | Myles PS, Buckland MR, Weeks AM, et al. Hemodynamic effects, myocardial ischemia, and timing of tracheal extubation with propofol-based anesthesia for cardiac surgery. Anesthesia and analgesia. 1997;84(1):12-19. |
| 55. Ohqvist 1985 | Ohqvist G, Settergren G. Ekestrom S. Brodin LA. The influence of isoflurane on blood flow in coronary bypass grafts. Acta anaesthesiologica Scandinavica. 1985;29(8):758-763. |
| 56. Ozer 2017 | Ozer E, Yilmaz R. Effect of different anesthetic techniques on mental outcome in elderly patients undergoing off-pump coronary artery bypass graft surgery. Turkiye Klinikleri Cardiovascular Sciences. 2017;29(1):17-22. |
| 57. Parker 2004 | Parker FC, Story DA, Poustie S, Liu G, McNicol L. Time to tracheal extubation after coronary artery surgery with isoflurane, sevoflurane, or target-controlled propofol anesthesia: a prospective, randomized, controlled trial. Journal of cardiothoracic and vascular anesthesia. 2004;18(5):613-619. |
| 58. Parsons 1994 | Parsons RS, Jones RM, Wrigley SR, MacLeod KG, Platt MW. Comparison of desflurane and fentanyl-based anaesthetic techniques for coronary artery bypass surgery. British journal of anaesthesia. 1994;72(4):430-438. |
| 59. Piriou 2007 | Piriou V, Mantz J, Goldfarb G, et al. Sevoflurane preconditioning at 1 MAC only provides limited protection in patients undergoing coronary artery bypass surgery: a randomized bi-centre trial. British journal of anaesthesia. 2007;99(5):624-631. |
| 60. Ramsay 1994 | Ramsay JG, DeLima LG, Wynands JE, O'Connor JP, Ralley FE, Robbins GR. Pure opioid versus opioid-volatile anesthesia for coronary artery bypass graft surgery: a prospective, randomized, double-blind study. Anesthesia and analgesia. 1994;78(5):867-875. |
| 61. Royse 2011 | Royse CF, Andrews DT, Newman SN, et al. The influence of propofol or desflurane on postoperative cognitive dysfunction in patients undergoing coronary artery bypass surgery. Anaesthesia. 2011;66(6):455-464. |
| 62. Shi 2007 | Shi Y. Study on brain cognitive dysfunction in perioperative period of off-pump coronary artery bypass grafting [Master]: Tianjin medical university;2007. |
| 63. Shi 2009 | Shi Y, Han J, Liu C. Effects of different anesthesia methods on postoperative cognitive function in off-pump coronary artery bypass grafting surgery. Tianjin medical journal. 2009;37(2):104-106. |
| 64. Shi 2019 | Shi Y, Wang W. Application of different anesthetic methods in coronary artery bypass grafting and the effect on postoperative outcome. Experimental and therapeutic medicine. 2019 January;17(1):695-700. |
| 65. Sirvinskas 2015 | Sirvinskas E, Kinderyte A, Trumbeckaite S, Lenkutis T, Raliene L, Giedraitis S, et al. Effects of sevoflurane vs. propofol on mitochondrial functional activity after ischemia-reperfusion injury and the influence on clinical parameters in patients undergoing CABG surgery with cardiopulmonary bypass. Perfusion (United Kingdom). 2015;30(7):590-595. |
| 66. Slogoff 1989 | Slogoff S, Keats AS. Randomized trial of primary anesthetic agents on outcome of coronary artery bypass operations. Anesthesiology. 1989;70(2):179-188. |
| 67. Soro 2012 | Soro M, Gallego L, Silva V, et al. Cardioprotective effect of sevoflurane and propofol during anaesthesia and the postoperative period in coronary bypass graft surgery: a double-blind randomised study. European journal of anaesthesiology. 2012;29(12):561-569. |
| 68. Story 2001 | [Story DA](https://www.ncbi.nlm.nih.gov/pubmed/?term=Story%20DA%5BAuthor%5D&cauthor=true&cauthor_uid=11605922), [Poustie S](https://www.ncbi.nlm.nih.gov/pubmed/?term=Poustie%20S%5BAuthor%5D&cauthor=true&cauthor_uid=11605922), [Liu G](https://www.ncbi.nlm.nih.gov/pubmed/?term=Liu%20G%5BAuthor%5D&cauthor=true&cauthor_uid=11605922), [McNicol PL](https://www.ncbi.nlm.nih.gov/pubmed/?term=McNicol%20PL%5BAuthor%5D&cauthor=true&cauthor_uid=11605922). Changes in plasma creatinine concentration after cardiac anesthesia with isoflurane, propofol, or sevoflurane: a randomized clinical trial. [Anesthesiology.](https://www.ncbi.nlm.nih.gov/pubmed/11605922) 2001;95(4):842-848. |
| 69. Sun 2016 | Sun P, Zhai W, Liu J, Er J, Han J. Effect of sevoflurane preconditioning on early postoperative cognitive function in patients undergoing off-pump coronary artery bypass grafting. International Journal of Anesthesiology and Resuscitation. 2016;37(7):587-591. |
| 70. Suryaprakash 2013 | Suryaprakash S, Chakravarthy M, Muniraju G, et al. Myocardial protection during off pump coronary artery bypass surgery: a comparison of inhalational anesthesia with sevoflurane or desflurane and total intravenous anesthesia. Annals of cardiac anaesthesia. 2013;16(1):4-8. |
| 71. Tempe 2011 | Tempe DK, Dutta D, Garg M, Minhas H, Tomar A, Virmani S. Myocardial protection with isoflurane during off-pump coronary artery bypass grafting: a randomized trial. Journal of cardiothoracic and vascular anesthesia. 2011;25(1):59-65. |
| 72. Tritapepe 2007 | Tritapepe L, Landoni G. Guarracino F, et al. Cardiac protection by volatile anaesthetics: a multicentre randomized controlled study in patients undergoing coronary artery bypass grafting with cardiopulmonary bypass. European journal of anaesthesiology. 2007;24(4):323-331. |
| 73. Urzua 1996 | [Urzua J](https://www.ncbi.nlm.nih.gov/pubmed/?term=Urzua%20J%5BAuthor%5D&cauthor=true&cauthor_uid=8909670), [Serra M](https://www.ncbi.nlm.nih.gov/pubmed/?term=Serra%20M%5BAuthor%5D&cauthor=true&cauthor_uid=8909670), [Lema G](https://www.ncbi.nlm.nih.gov/pubmed/?term=Lema%20G%5BAuthor%5D&cauthor=true&cauthor_uid=8909670), [Canessa R](https://www.ncbi.nlm.nih.gov/pubmed/?term=Canessa%20R%5BAuthor%5D&cauthor=true&cauthor_uid=8909670), [Gonzalez R](https://www.ncbi.nlm.nih.gov/pubmed/?term=Gonzalez%20R%5BAuthor%5D&cauthor=true&cauthor_uid=8909670), [Meneses G](https://www.ncbi.nlm.nih.gov/pubmed/?term=Meneses%20G%5BAuthor%5D&cauthor=true&cauthor_uid=8909670), et al. Comparison of isoflurane, halothane and fentanyl in patients with decreased ejection fraction undergoing coronary surgery. [Anaesth Intensive Care.](https://www.ncbi.nlm.nih.gov/pubmed/8909670) 1996;24(5):579-584. |
| 74. Wang 2004 | Wang X, Jarvinen O. Kuukasjarvi P, et al. Isoflurane produces only minor preconditioning in coronary artery bypass grafting. Scandinavian cardiovascular journal : SCJ. 2004;38(5):287-292. |
| 75. Wang 2009 | Wang C, Xu Y, Lu J, Qing E. Study on hemodynamics and myocardial effects of sevoflurane combined with sufentanil anesthesia in coronary artery bypass grafting without stopping. Journal of cardiovascular and pulmonary diseases. 2009;28(4):220-235. |
| 76. Wang 2012 | Wang Y. Clinical study on the effect of sevoflurane lavage with extracorporeal circulation bypass on myocardial injury in patients with coronary artery bypass grafting [m]: Tianjin medical university; 2012. |
| 77. Wang 2017 | Wang X. Relationship between anesthetic factors and myocardial injury in patients undergoing cardiopulmonary bypass: dexmedetomidine combined with sevoflurane anesthesia. Chinese journal of anesthesiology. 2017;37(5). |
| 78. Wasowicz 2018 | Wasowicz M, Jerath A, Luksun W, Sharma V, Mitsakakis N, Meineri M, et al. Comparison of propofol-based versus volatile-based anaesthesia and postoperative sedation in cardiac surgical patients: A prospective, randomized, study. Anaesthesiology intensive therapy. 2018;50(3):200-209. |
| 79. Woodcock 1987 | Woodcock TE, Murkin JM, Farrar JK, et al. Pharmacologic EEG suppression during cardiopulmonary bypass: cerebral hemodynamic and metabolic effects of thiopental or isoflurane during hypothermia and normothermia. Anesthesiology. 1987;67(2):218-224. |
| 80. Xia 2006 | Xia Z, Huang Z. Ansley DM. Large-dose propofol during cardiopulmonary bypass decreases biochemical markers of myocardial injury in coronary surgery patients: a comparison with isoflurane. Anesthesia and analgesia. 2006;103(3):527-532. |
| 81. Yan 2012 | Yan K. Clinical study on myocardial protective effect of sevoflurane post-treatment on patients with non-extracorporeal coronary artery bypass grafting [m]: Central south university; 2012. |
| 82. Yang 2012 | Yang Y, Zhang J, Zheng X, Zhang J. Effect of sevoflurane post-treatment on cTnI level in patients with diabetes mellitus and coronary heart disease. Chinese journal of laboratory diagnosis. 2012;16(8):1417-1419. |
| 83. Yi 2018 | Yi L, Yao S, Chen X, Wu Q, Huang M, Wang T. Effects of anesthetic drugs on postoperative cognitive function of patients undergoing off-pump coronary artery bypass grafting. Medical Journal of Wuhan University. 2018;39(4):627-631. |
| 84. Yildirim 2009 | Yildirim V, Doganci S, Aydin A, Bolcal C, Demirkilic U, Cosar A. Cardioprotective effects of sevoflurane, isoflurane, and propofol in coronary surgery patients: a randomized controlled study. Heart surgery forum. 2009;12(1). |
| 85. Yu 2008 | Yu C, Beattie WS, Huang Y, Luo A. Protective effect of sevoflurane pretreatment on perioperative myocardium of patients undergoing coronary artery bypass grafting with extracorporeal circulation. Chinese journal of anesthesiology. 2007;27(6):508-511. |
| 86. Zhang 2019 | Zhang L, Wang CB, Li B, Lin DM, Ma J. RhoA/rho-kinase, nitric oxide and inflammatory response in LIMA during OPCABG with isoflurane preconditioning. Journal of cardiothoracic surgery. 2019;14(1). |
| 87. Zhang 2019b | Zhang T, Jiang H, An M, Zhang L, Yang J, Chai J, et al. Effects of different anesthesia schemes on stress and wake quality of coronary artery bypass grafting patients. Chinese Heart Journal. 2019;31(01):34-53. |
| 88. Zhao 2013 | Zhao Y, Shi H, Miao J, Wang Z, Ge Y, Wei H, et al. Effects of sevoflurane pretreatment on left ventricular function in patients undergoing coronary artery bypass grafting with extracorporeal circulation. Chinese Journal of Anesthesiology. 2013;33(12):1423-1426. |
| 89. Zou 2010 | Zou H, Wu L, Zhou Q, Cheng B. Different anesthesia method for extracorporeal circulation the influence of early postoperative cognitive function in patients with coronary artery bypass graft. Modern Journal of integrated traditional Chinese and western medicine. 2010;19(4):391-410. |
