# Supplementary material for: Volatile anesthetics versus total intravenous anesthesia in patients undergoing coronary artery bypass grafting: An updated meta-analysis and trial sequential analysis of randomized controlled trials
Source: PLoS One. 2019 Oct 29;14(10):e0224562. doi: 10.1371/journal.pone.0224562 (PMC6818786; doi:10.1371/journal.pone.0224562)
Supplement: S4 Table — (DOCX) [file pone.0224562.s004.docx]

**S4 Table. Characteristics of the included studies**

| **Study** | **Country** | **Mean age (volatile/TIVA)** | **Male (volatile/TIVA, %)** | **CABG type** | **Case (volatile/TIVA, n)** | **Volatile agent** | **TIVA agent** | **Longest follow-up** |
| --- | --- | --- | --- | --- | --- | --- | --- | --- |
| Amr 2010 | Egypt | 53±7 / 55±6 | 80/86.67 | On-Pump | 15/15 | Isoflurane | Sufentanil, midazolam | 1 year |
| Azab 2000 | Netherland | — | — | On-Pump | 15/15 | Sevofurane | Sufentanil, midazolam | Hospital stay |
| Azab 2003 | Netherland | 60±10 / 63±8 | 70/80 | On-Pump | 10/10 | Sevoflurane | Sufentanil, midazolam | Hospital stay |
| Baki 2013 | Turkey | 64.57±10.84 / 66.45±13.04 | 60/55 | On-Pump | 20/20 | Desflurane | Propofol | ICU stay |
| Ballester 2011 | Spain | 62.44±10.86 / 59.65±7.60 | 78/95 | Off-pump | 18/20 | Sevoflurane | Propofol | Hospital stay |
| Bein 2005 | Germany | 63±10 / 60±13 | 50/57.69 | Off-pump | 24/26 | Sevoflurane | Propofol | Hospital stay |
| Bein 2008 | Germany | 66.5±5.97 / 62±7 | 79.17/71.43 | On-Pump | 24/14 | Sevoflurane | Propofol | Hospital stay |
| Belhomme 1999 | Japan | 68±9 / 70±9 | 90/90 | On-Pump | 10/10 | Isoflurane | Fentanyl, flunitrazepam, pancuronium | 72 hours |
| Bharti 2008 | India | 54±7.67 / 54.4±7.55 | 86.67/135 | On-Pump | 15/15 | Sevoflurane | Propofol | Hospital stay |
| Bi 2008 | China | 49.1±13.5 / 53.7±10.6 | 75/80 | On-Pump | 40/40 | Isoflurane | Propofol | 1～10 days |
| Botero 2000 | America | 62±9 / 63±2 | 68.29/72.22 | On-Pump | 41/36 | Enflurane | Propofol, ketamine | Hospital stay |
| Chang 2007 | China | 65±1.3 / 63±1.1 | 68/56 | Off-pump | 25/25 | Isoflurane | Fentanyl, propofol | ICU stay |
| Chao 2010 | China | 62.47±6.3 / 60.2±6.24 | 92.30/86.67 | Off-pump | 13/15 | Isoflurane | Propofol | 4 days |
| Chen 2014 | China | 62.13±8.27 / 63.23±7.89 | — | On-Pump | 15/15 | Sevoflurane | Propofol | ICU stay |
| Conzen 2003 | Germany | 62±9 / 65±8 | 80/60 | Off-pump | 10/10 | Sevoflurane | Propofol | Hospital stay |
| De Hert 2003 | Belgium | 74.5±2.99 / 76±4 | 86.68/86.67 | On-Pump | 30/15 | Desflurane, Sevoflurane | Propofol | 36 hours |
| De Hert 2004a | Belgium | 67±9.25 / 66.5±8.99 | 81.88/80.63 | On-Pump | 160/160 | Sevoflurane, desflurane | Propofol, midazolam | Hospital stay |
| De Hert 2004b | Belgium | 65.67±10.32 / 66±10 | 80/80 | On-Pump | 150/50 | Sevoflurane | Propofol | 30 days |
| De Hert 2009 | Belgium | 67.01±9.03 / 68±9 | 81.78/80.69 | On-Pump | 269/145 | Sevoflurane, desflurane | Any intravenous agent | 1 year |
| Drenger 2008 | Israel | 69±10 / 66±10 | 75/75 | off-pump | 8/8 | Enflurane | Fentanyl, midazolam, pancuronium bromide | Hospital stay |
| Driessen 1997 | Belgium | 63±7 / 61±4 | — | On-Pump | 15/15 | Isoflurane | Midazolam | ICU stay |
| Flier 2010 | Turkey | 66 / 67 | 80.5/76.7 | On-Pump | 41/43 | Isoflurane | Propofol, sufentanil | 1 year |
| Frassdorf 2009 | UK | 66±8.35 / 66±8 | 90/90 | On-Pump | 20/10 | Sevoflurane | Propofol, sufentanil, pancuronim bromide | Hospital stay |
| Garcia 2005 | Switzerland | 62±10 / 65±10 | 83.78/80 | On-Pump | 37/35 | Sevoflurane | Propofol, etomidate | 1 year |
| Gravel 1999 | Canada | 60±9 / 63±9 | 66.67/73.33 | On-Pump or off-pump | 15/15 | Sevoflurane | Midazolam, propofol | Hospital stay |
| Guarracino 2006 | Italy | 69±9 / 69±8 | 86/78.2 | Off-pump | 56/54 | Desflurane | Propofol | 30 days |
| Hall 1991 | Canada | 57±8.2 / 58±9.8 | 100/75 | On-Pump | 23/24 | Enflurane | Propofol, sufentanil | Hospital stay |
| Hall 1993 | Canada | 58.3±8.3 / 61.2±8 | 94.44/90.48 | On-Pump | 18/21 | Enflurane | Propofol | Hospital stay |
| Haroun-Bizri 2001 | Lebanon | 57.1±8.6 / 60.9±10.3 | 78.57/76.19 | On-Pump | 28/21 | Isoflurane | Thiopental, midazolam, sufentanil, cisatracurium | Hospital stay |
| Helman 1992 | America | 63±9 / 63±9 | 88/84 | On-Pump | 100/100 | Desflurane | Sufentanil | 3 days |
| Hofland 2017 | Netherland | 64±9 / 64±9 | 85/88 | On-Pump | 165/166 | Sevoflurane | Propofol | Hospital stay or 30 days |
| Huang 2011 | China | 59.8±10.3 / 63.1±6.4 | 83.38/83.33 | On-Pump | 30/30 | Isoflurane | Propofol | Hospital stay |
| Jerath 2015 | Canada | 65±9 / 63±10 | 91.04/94.59 | On-Pump | 67/74 | Isoflurane, sevoflurane | Propofol | Hospital stay |
| Ji 2013 | China | 61.61±9.48 / 61.94±10.36 | 67.86/71.43 | Off-pump | 28/28 | Sevoflurane | Propofol | Hospital stay |
| Jia 2015 | China | 61±8 / 62±8 | — | Off-pump | 33/34 | Sevoflurane | Propofol | Hospital stay |
| JIa 2018 | China | 67.21±4.77 / 67.78±4.70 | 65/62.5 | — | 40/40 | Sevoflurane | Propofol | 4 days |
| Julier 2003 | Switzerland | 62±10 / 65±10 | 83.78/80 | On-Pump | 37/35 | Sevoflurane | Propofol, etomidate | Hospital stay |
| Kendall 2004 | UK | 58.1±7 / 68.1±11.1 | 70/70 | Off-pump | 10/10 | Isoflurane | Propofol | 48 hours |
| Kim 2011 | Korea | 64±11 / 65±9 | 68.08/59.57 | Off-pump | 47/47 | Sevoflurane | Propofol | Hospital stay |
| Landoni 2019 | International | 62.2±8.37 / 62.3±8.41 | 80.8/81.5 | On-Pump or off-pump | 2709/2691 | Volatile anesthetic (desflurane, isoflurane, sevoflurane) | Any intravenous agent | 1 year |
| Law-Koune 2006 | France | 64.8±9.9 / 59.7±4.8 | 88.89/88.89 | Off-pump | 9/9 | Sevoflurane | Propofol | Hospital stay |
| Lee 2006 | China | 68.0±10.5 / 66.0±10.1 | 80/85 | On-Pump | 20/20 | Isoflurane | Diazepam, fentanyl, pancuronium | Hospital stay |
| Lemoine 2018 | France | 68±11 / 68±10 | 79/62 | On-Pump | 24/21 | Sevoflurane | Propofol | Hospital stay |
| Leung 1991 | America | 61±9 / 62±8 | — | On-Pump | 62/124 | Isoflurane | Sufentanil | 3 days |
| Li 2010 | China | — | — | Off-pump | 15/15 | Isoflurane | Propofol | 5 days |
| Likhvantsev 2016 | Russia | 62±8 / 61±8 | 87/89 | On-Pump | 437/431 | Sevoflurane | Propofol | 30 days |
| Liu 2012 | China | 61.8±6.69 / 58.87±11.10 | 73.33/86.67 | Off-pump | 15/15 | Sevoflurane | Propofol | Hospital stay |
| Lorsomradee 2006 | Belgium | 67±9 / 66±10 | 85.63/75 | On-Pump | 160/160 | Sevoflurane | Propofol | Hospital stay |
| Lu 2003 | China | 67±1.2 / 68±1 | 75.93/62.26 | On-Pump | 54/53 | Isoflurane | Fentanyl | Hospital stay |
| Luo 2016 | China |  | — | Off-pump | 30/30 | Sevoflurane | Propofol | Hospital stay |
| Meco 2007 | Italy | 59.09±6.5 / 61.9±3.1 | 64.28/71.43 | On-Pump | 14/14 | Desflurane | Fentanyl, midazolam, propofol, pancuronium | 72 hours |
| Mora 1995 | Georgia | 64±9 / 62.99±8.26 | 16.67/18.18 | On-Pump | 24/66 | Enflurane | Fentanyl, propofol, thiopental | Hospital stay |
| Mrozinski 2014 | Poland | 66/59.5 | 65.63/78.57 | Off-pump | 32/28 | Desflurane | Propofol | Hospital stay or 30 days |
| Myles 1997 | Australia | 65±10 / 63±11 | 71.21/89.66 | On-Pump | 66/58 | Enflurane | Propofol | Hospital stay |
| Ohqvist 1985 | Sweden | 54±3 / 63±2 | 80/80 | On-Pump | 10/10 | Isoflurane | Fentanyl | Hospital stay |
| Ozer 2017 | Turkey | 69±3 / 66±4 | 55/57.5 | Off-pump | 40/40 | Isoflurane | Propofol | Hospital stay |
| Parker 2004 | Australia | 66.5/65 | 80.93/83.05 | On-Pump | 236/118 | Isoflurane, Sevoflurane | Propofol | ICU stay |
| Parsons 1994 | UK | 58.5/59.7 | 76.92/84 | On-Pump | 26/25 | Desflurane | Fentanyl | Hospital stay |
| Piriou 2007 | France | 63/61 | — | On-Pump | 36/36 | Sevoflurane | Propofol | Hospital stay |
| Ramsay 1994 | America | 59.5±2.04 / 60±2 | 84/88 | On-Pump | 50/25 | Isoflurane, enflurane | Sufentanil | Hospital stay |
| Royse 2011 | Australia | 61.8±10.4 / 63.8±10.6 | 80/90 | On-Pump | 91/89 | Desflurane | Propofol | Hospital stay |
| Shi 2007 | China | 63.8±6.4 / 64.5±6.3 | 66.7/60 | Off-pump | 15/15 | Isoflurane | Propofol | Hospital stay |
| Shi 2009 | China | 63.8±6.4 / 64.5±6.3 | 66.7/60 | Off-pump | 15/15 | Isoflurane | Propofol | 7 days |
| Shi 2019 | China | 20-38 / 20-40 | 61.70/57.45 |  | 47/47 | Sevoflurane | Fentanyl, propofol | Hospital stay |
| Sirvinskas 2015 | Lithuania | 66.1±8.4 / 67.4±7.7 | 75/80.6 | On-Pump | 36/36 | Sevoflurane | Propofol | Hospital stay |
| Slogoff 1989 | America | 58.5/59.8 | 83.5/82.3 | On-Pump | 758/254 | Enflurane, halothane, isoflurane | Sufentanil | Hospital stay |
| Soro 2012 | Spain | 68.3±10.7 / 69.4±9.3 | 75/81.1 | On-Pump | 36/37 | Sevoflurane | Propofol | Hospital stay |
| Story 2001 | Australia | 66.5/65 | 80.93/83.05 | On-Pump | 236/118 | Isoflurane, sevoflurane | Propofol | 3 days |
| Sun 2016 | China | 62±7 / 61±6 | 73.33/76.67 | Off-pump | 30/30 | Sevoflurane | Propofol | Hospital stay |
| Suryaprakash 2013 | India | 62.73±8.19 / 61.03±8.09 | 93/92.31 | Off-pump | 100/39 | Sevoflurane, desflurane | Propofol | Hospital stay |
| Tempe 2011 | India | 53±8 / 54±9 | 100/85 | Off-pump | 20/20 | Isoflurane | Propofol | 7 days |
| Tritapepe 2007 | Italy | 64±9.6 / 66±9.2 | 84/80 | On-Pump | 75/75 | Desflurane | Propofol | ICU stay |
| Urzua 1996 | Chile | 58±8.59 / 55±8.7 | — | On-Pump | 18/7 | Halothane, isoflurane | Fentanyl | Hospital stay |
| Wang 2004 | Finland | 61.7±8 / 63.6±9.6 | 100/93.75 | On-Pump | 18/16 | Isoflurane | Propofol, sufentanil | Hospital stay |
| Wang 2009 | China | — | — | Off-pump | 30/30 | Sevoflurane | Propofol | Hospital stay |
| Wang 2012 | China | 60±8 / 59±7 | 85/75 | On-Pump | 20/20 | Sevoflurane | Midazolam, etomidate, sufentanil, propofol, rocuronium bromide | Hospital stay |
| Wang 2017 | China | 61.5±7.01 / 63±6.1 | 63.64/56.82 | On-Pump | 44/44 | Sevoflurane | Dexmedetomidine | 24 hours |
| Wasowicz 2018 | Canada | 65±9 / 63±10 | 90/94 | On-Pump | 60/67 | Isoflurane, sevoflurane | Propofol | Hospital stay |
| Woodcock 1987 | UK | 55.5±9.9 / 54.83±9.81 | 91.67/89.47 | On-Pump | 12/19 | Isoflurane | Fentanyl, thiopental | Hospital stay |
| Xia 2006 | Canada | 65±4 / 64±4.9 | 66.67/69.44 | On-Pump | 18/36 | Isoflurane | Propofol | Hospital stay |
| Yan 2012 | China | 60.3±5.94 / 60.67±4.08 | 91.67/91.67 | Off-pump | 24/12 | Sevoflurane | Midazolam, sufentanil, propofol, cisatracurium | Hospital stay |
| Yang 2012 | China | 59.2±3.01 / 57.3±3.71 | 86.67/80 | On-Pump | 15/15 | Sevoflurane | Midazolam, etomidate, fentanyl, propofol, vecuronium bromide | Hospital stay |
| Yi 2018 | China | 63.8±6.77 / 60.69±7.31 | 76.67/81.67 | Off-pump | 30/60 | Sevoflurane | Propofol, midazolam | 7 days |
| Yildirim 2009 | Turkey | 68.5±9.93 / 67±10 | 77.55/70 | On-Pump | 40/20 | Isoflurane, Sevoflurane | Propofol | 30 days |
| Yu 2008 | China | 58±10 / 59±9 | 70/75 | On-Pump | 20/20 | Sevoflurane | Propofol | Hospital stay |
| Zhang 2019 | China | 57.5±5.2 / 57.3±8.2 | 80/66.67 | Off-pump | 15/15 | Isoflurane | Propofol | Hospital stay |
| Zhang 2019b | China | 66.5±4,52 / 65±4 | 70/63.33 | On-Pump | 60/30 | Sevoflurane | Sufentanil, propofol | Hospital stay |
| Zhao 2013 | China | 65±8 / 63±8 | 73.33/86.67 | On-Pump | 15/15 | Sevoflurane | Midazolam, sufentanil, propofol, rocuronium bromide | ICU stay |
| Zou 2010 | China | 56±12 / 55±10 | 73.33/70 | Off-pump | 30/30 | Sevoflurane | Propofol | 24 hours |

TIVA, total intravenous anesthesia; CABG, coronary artery bypass; ICU, intensive care unit.
