# Supplementary material for: Volatile anesthetics versus total intravenous anesthesia in patients undergoing coronary artery bypass grafting: An updated meta-analysis and trial sequential analysis of randomized controlled trials
Source: PLoS One. 2019 Oct 29;14(10):e0224562. doi: 10.1371/journal.pone.0224562 (PMC6818786; doi:10.1371/journal.pone.0224562)
Supplement: S5 Table — (DOCX) [file pone.0224562.s005.docx]

**S5 Table. Grade evidence profile for each outcome**

| **Quality assessment** | | | | | | | **No of patients** | | **Effect** | | **Quality** | **Importance** |
| --- | --- | --- | --- | --- | --- | --- | --- | --- | --- | --- | --- | --- |
|  |  |  |  |  |  |  |  |  |  |  |  |  |
| **No of studies** | **Design** | **Risk of bias** | **Inconsistency** | **Indirectness** | **Imprecision** | **Other considerations** | **Volatile anesthetics** | **TIVA** | **Relative (95% CI)** | **Absolute** |  |  |
| **Operative mortality** | | | | | | | | | | | | |
| 44 | randomised trials | no serious risk of bias | no serious inconsistency | no serious indirectness | serious^1^ | none | 83/6206  (1.3%) | 80/5361  (1.5%) | RR 0.92 (0.68 to 1.24) | 1 fewer per 1000 (from 5 fewer to 4 more) | ⊕⊕⊕O MODERATE | CRITICAL |
| **One-year mortality** | | | | | | | | | | | | |
| 5 | randomised trials | no serious risk of bias | serious^2^ | no serious indirectness | serious^1^ | none | 89/3047  (2.9%) | 100/2906  (3.4%) | RR 0.64 (0.32 to 1.26) | 12 fewer per 1000 (from 23 fewer to 9 more) | ⊕⊕OO LOW | CRITICAL |
| **Length of stay in ICU** | | | | | | | | | | | | |
| 43 | randomised trials | serious^3^ | very serious^2^ | no serious indirectness | serious^1^ | none | 4545 | 4435 | - | MD 4.14 lower (5.63 to 2.66 lower) | ⊕OOO VERY LOW | IMPORTANT |
| **Length of stay in hospital** | | | | | | | | | | | | |
| 34 | randomised trials | serious^3^ | serious^2^ | no serious indirectness | no serious imprecision | none | 4718 | 4549 | - | MD 1.22 lower (1.81 to 0.62 lower) | ⊕⊕OO LOW | IMPORTANT |
| **Myocardial infarction** | | | | | | | | | | | | |
| 28 | randomised trials | no serious risk of bias | no serious inconsistency | no serious indirectness | no serious imprecision | none | 115/4365  (2.6%) | 121/4209  (2.9%) | RR 0.94 (0.73 to 1.21) | 2 fewer per 1000 (from 8 fewer to 6 more) | ⊕⊕⊕⊕ HIGH | IMPORTANT |
| **Heart failure** | | | | | | | | | | | | |
| 4 | randomised trials | no serious risk of bias | no serious inconsistency | no serious indirectness | serious^1^ | none | 1/201  (0.5%) | 6/260  (2.3%) | RR 0.39 (0.08 to 2.01) | 14 fewer per 1000 (from 21 fewer to 23 more) | ⊕⊕⊕O MODERATE | IMPORTANT |
| **Arrhythmia** | | | | | | | | | | | | |
| 29 | randomised trials | no serious risk of bias | no serious inconsistency | no serious indirectness | no serious imprecision | none | 259/1707  (15.2%) | 244/1377  (17.7%) | RR 0.89 (0.77 to 1.03) | 19 fewer per 1000 (from 41 fewer to 5 more) | ⊕⊕⊕⊕ HIGH | IMPORTANT |
| **Stroke** | | | | | | | | | | | | |
| 2 | randomised trials | no serious risk of bias | no serious inconsistency | no serious indirectness | serious^1^ | none | 22/2682  (0.8%) | 15/2671  (0.6%) | RR 1.46 (0.76 to 2.81) | 3 more per 1000 (from 1 fewer to 10 more) | ⊕⊕⊕O MODERATE | IMPORTANT |
| **Delirium** | | | | | | | | | | | | |
| 3 | randomised trials | serious^3^ | no serious inconsistency | no serious indirectness | no serious imprecision | none | 78/2780  (2.8%) | 88/2802  (3.1%) | RR 0.96 (0.71 to 1.29) | 1 fewer per 1000 (from 9 fewer to 9 more) | ⊕⊕⊕O MODERATE | IMPORTANT |
| **Postoperative cognitive impairment** | | | | | | | | | | | | |
| 8 | randomised trials | serious^3^ | serious^2^ | no serious indirectness | no serious imprecision | none | 106/2897  (3.7%) | 111/2895  (3.8%) | RR 1.2 (0.74 to 1.94) | 8 more per 1000 (from 10 fewer to 36 more) | ⊕⊕OO LOW | IMPORTANT |
| **Acute kidney injury** | | | | | | | | | | | | |
| 5 | randomised trials | no serious risk of bias | no serious inconsistency | no serious indirectness | no serious imprecision | none | 160/2868  (5.6%) | 158/2838  (5.6%) | RR 0.98 (0.79 to 1.22) | 1 fewer per 1000 (from 12 fewer to 12 more) | ⊕⊕⊕⊕ HIGH | IMPORTANT |
| **The use of IABP** | | | | | | | | | | | | |
| 6 | randomised trials | no serious risk of bias | no serious inconsistency | no serious indirectness | serious^1^ | none | 7/2769  (0.3%) | 14/2818  (0.5%) | RR 0.67 (0.29 to 1.54) | 2 fewer per 1000 (from 4 fewer to 3 more) | ⊕⊕⊕O MODERATE | IMPORTANT |
| **The use of other mechanical circulatory support** | | | | | | | | | | | | |
| 4 | randomised trials | no serious risk of bias | serious^4^ | no serious indirectness | no serious imprecision | none | 10/2744  (0.4%) | 12/2714  (0.4%) | RR 0.73 (0.33 to 1.63) | 1 fewer per 1000 (from 3 fewer to 3 more) | ⊕⊕⊕O MODERATE | IMPORTANT |

TIVA, total intravenous anesthesia; CI: confidence interval; RR: risk ratio; ICU, intensive care unit; IABP, intra-aortic balloon pump.

^1^ Imprecisions revealed by trial sequential analysis (TSA)
^2^ Significant heterogeneity
^3^ Lack of or unclear blinding
^4^ Large differences between point estimates
