# Supplementary figures and images for: Volatile anesthetics versus total intravenous anesthesia in patients undergoing coronary artery bypass grafting: An updated meta-analysis and trial sequential analysis of randomized controlled trials
Source: PLoS One. 2019 Oct 29;14(10):e0224562. doi: 10.1371/journal.pone.0224562 (PMC6818786; doi:10.1371/journal.pone.0224562)

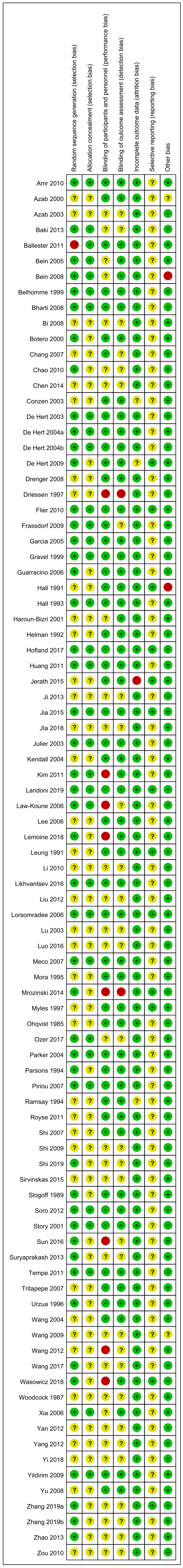

Supplement: S1 Fig — (TIF) [file pone.0224562.s006.tif]

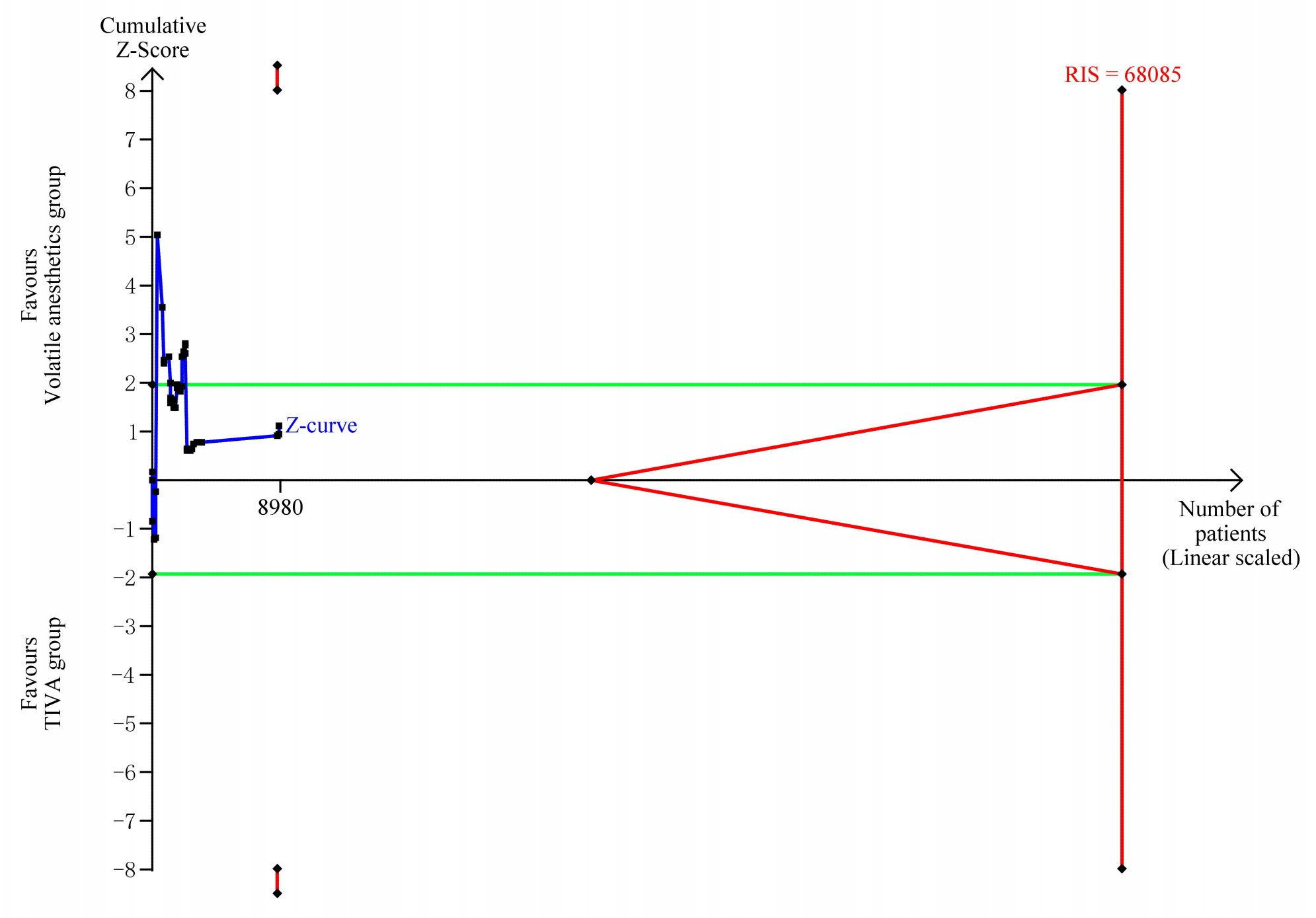

Supplement: S2 Fig — The risk of type Ⅰ error was set at 5% with a power of 80%. The variance was calculated from the data obtained from the included trials. The mean difference reduction was set at -0.05 h. (TIF) [file pone.0224562.s007.tif]

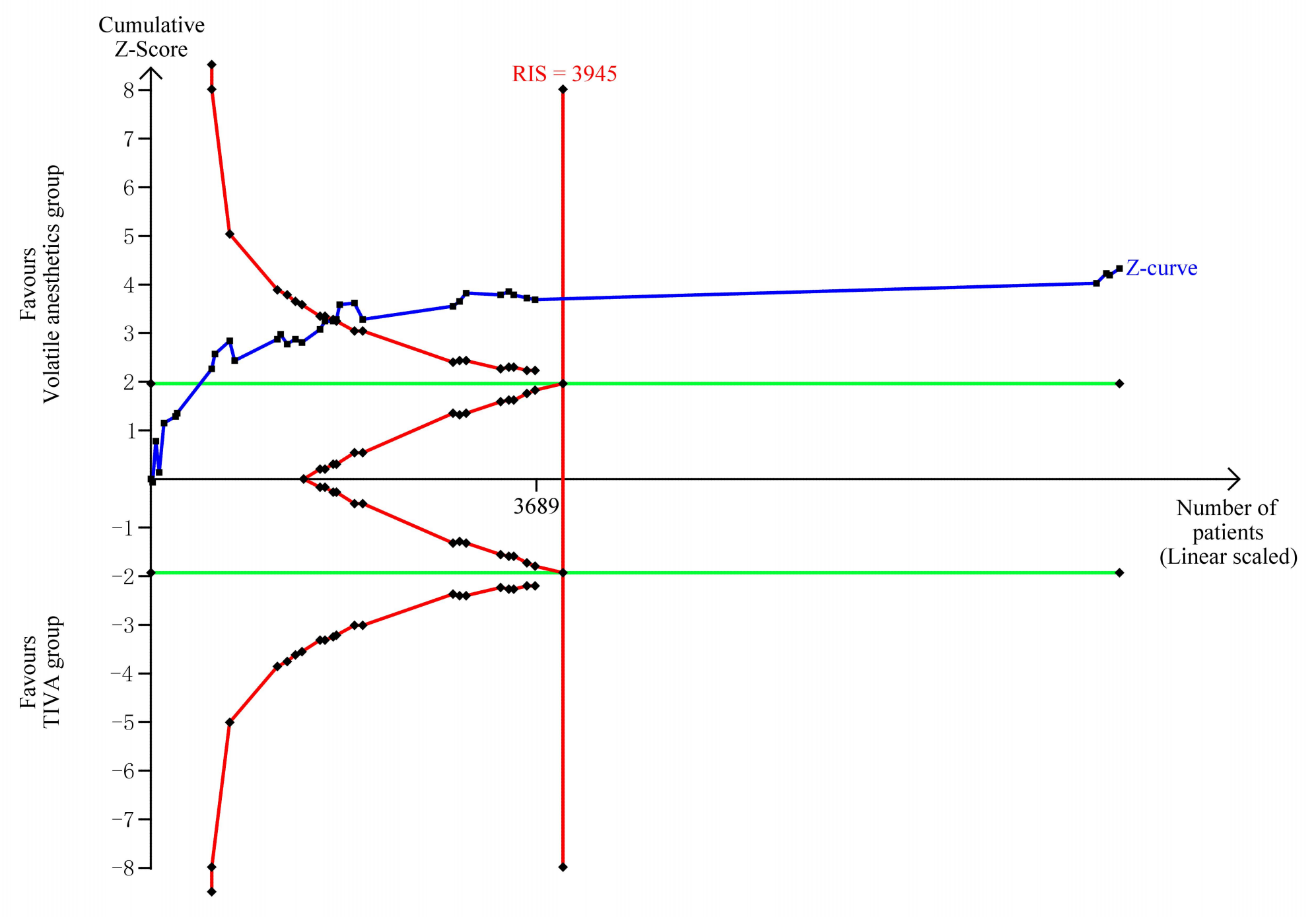

Supplement: S3 Fig — The risk of type Ⅰ error was set at 5% with a power of 80%. The variance was calculated from the data obtained from the included trials. The relative risk reduction (RRR) was set at 20%. (TIF) [file pone.0224562.s008.tif]

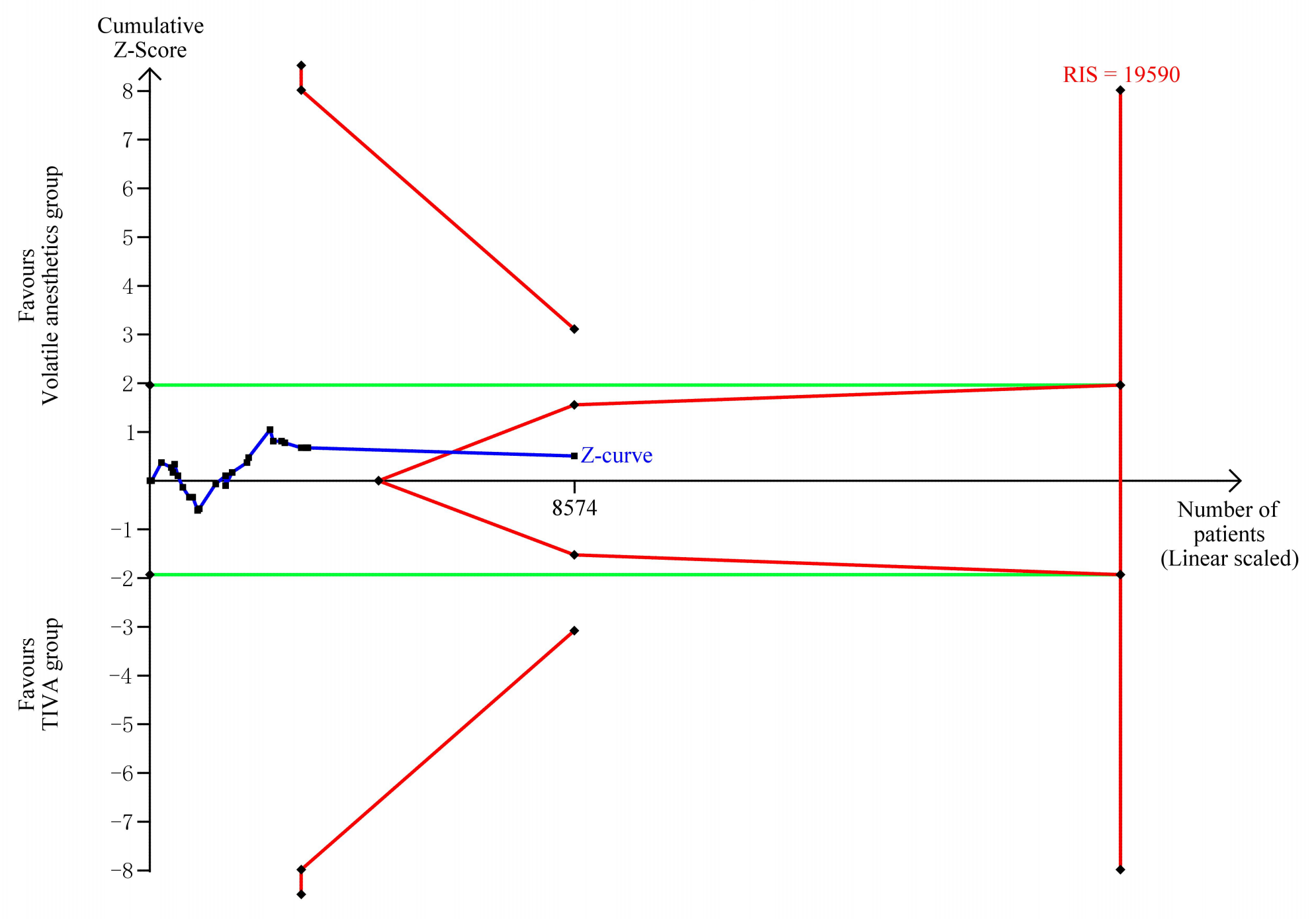

Supplement: S4 Fig — The risk of type Ⅰ error was set at 5% with a power of 80%. The variance was calculated from the data obtained from the included trials. The mean difference reduction was set at -1.18 d. (TIF) [file pone.0224562.s009.tif]

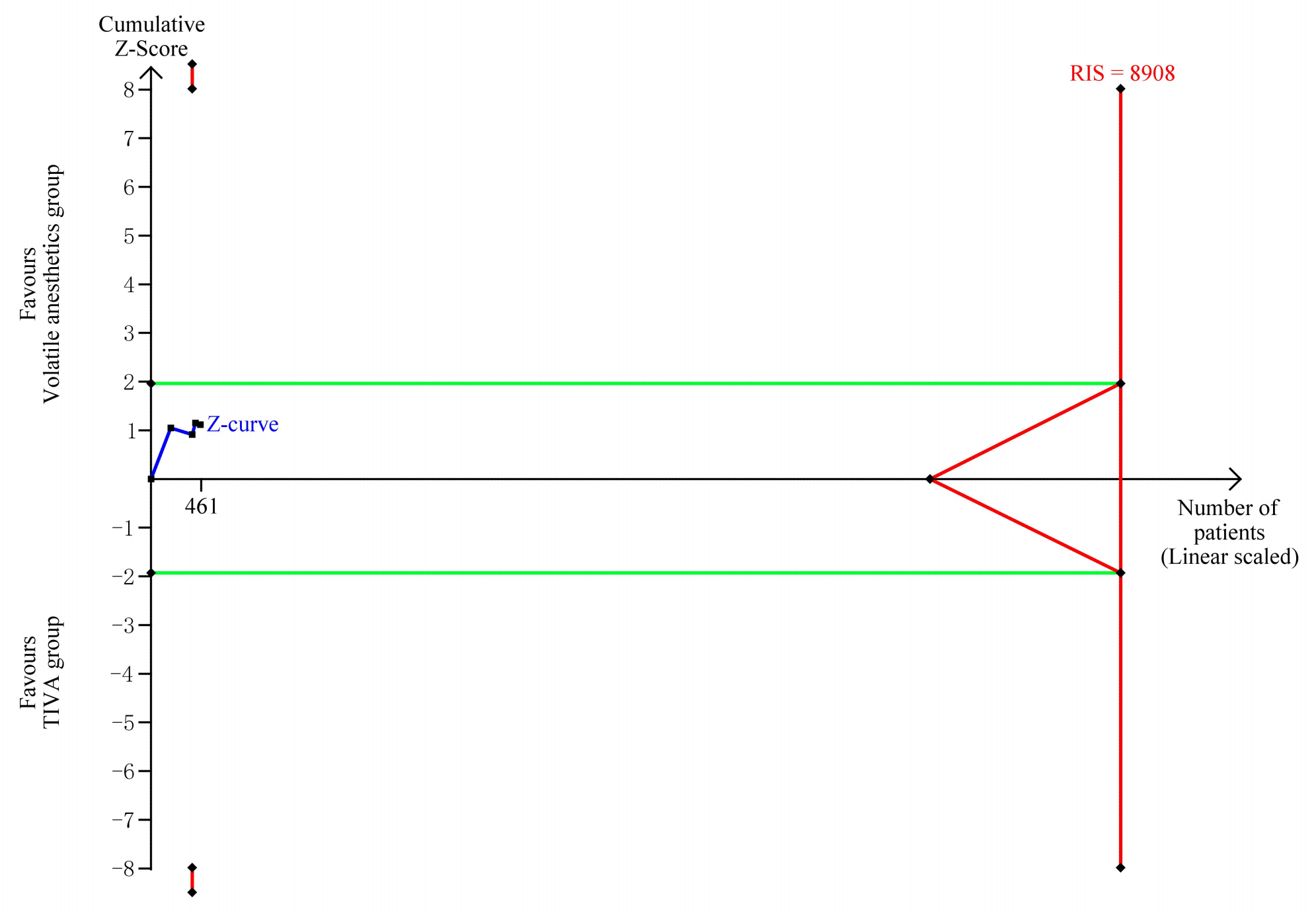

Supplement: S5 Fig — The risk of type Ⅰ error was set at 5% with a power of 80%. The variance was calculated from the data obtained from the included trials. The relative risk reduction (RRR) was set at 30%. (TIF) [file pone.0224562.s010.tif]

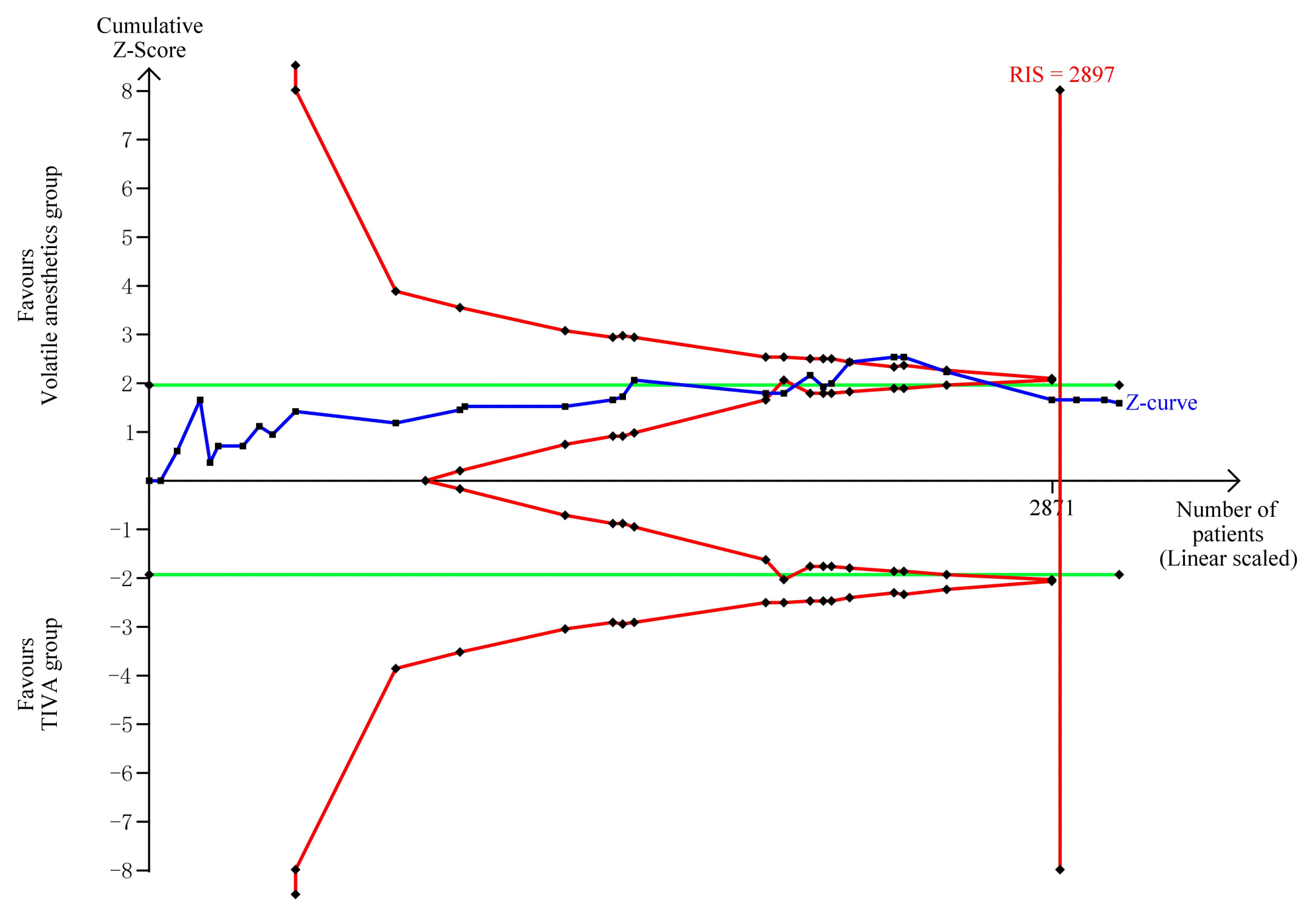

Supplement: S6 Fig — The risk of type Ⅰ error was set at 5% with a power of 80%. The variance was calculated from the data obtained from the included trials. The relative risk reduction (RRR) was set at 20%. (TIF) [file pone.0224562.s011.tif]

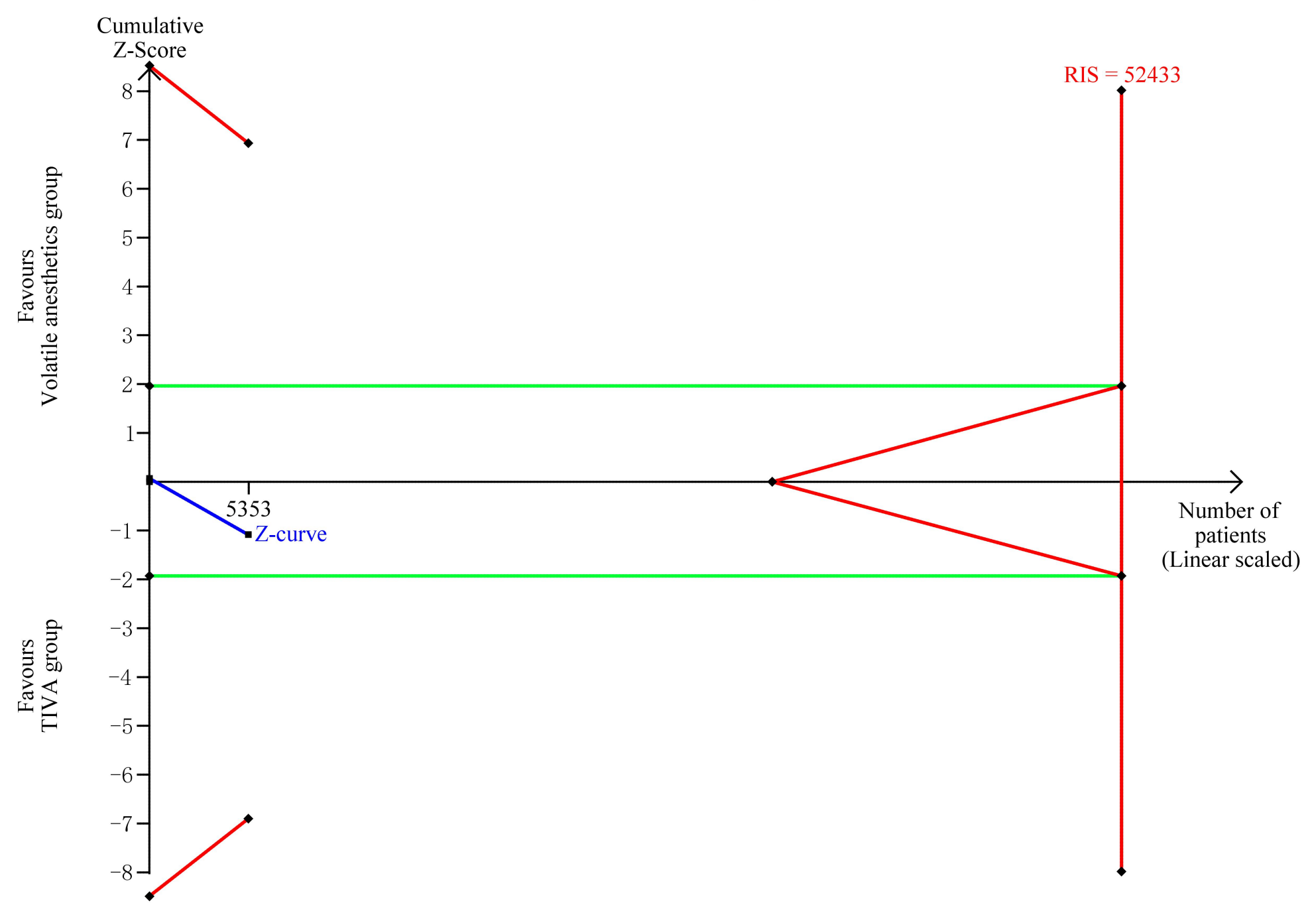

Supplement: S7 Fig — The risk of type Ⅰ error was set at 5% with a power of 80%. The variance was calculated from the data obtained from the included trials. The relative risk reduction (RRR) was set at 30%. (TIF) [file pone.0224562.s012.tif]

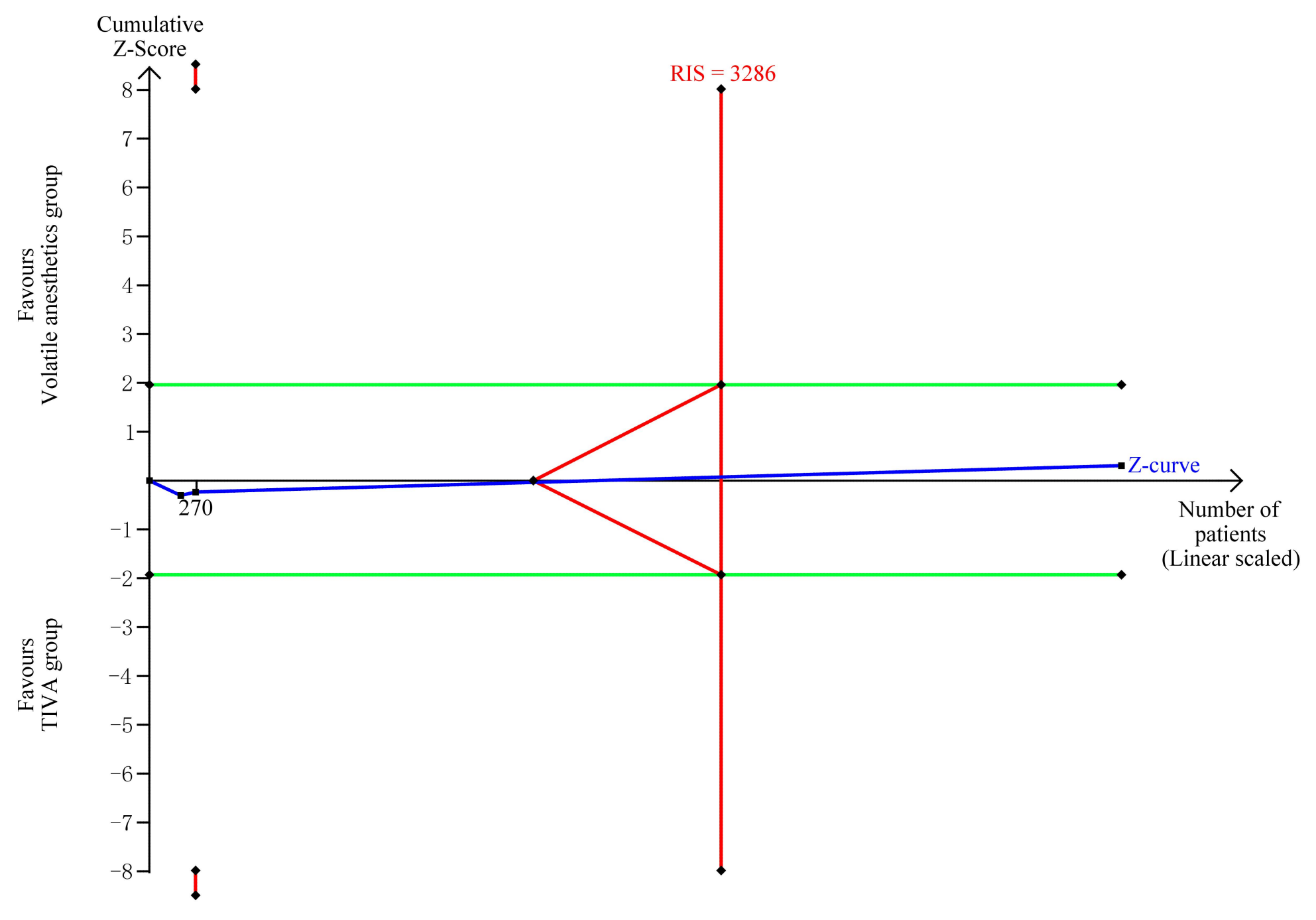

Supplement: S8 Fig — The risk of type Ⅰ error was set at 5% with a power of 80%. The variance was calculated from the data obtained from the included trials. The relative risk reduction (RRR) was set at 20%. (TIF) [file pone.0224562.s013.tif]

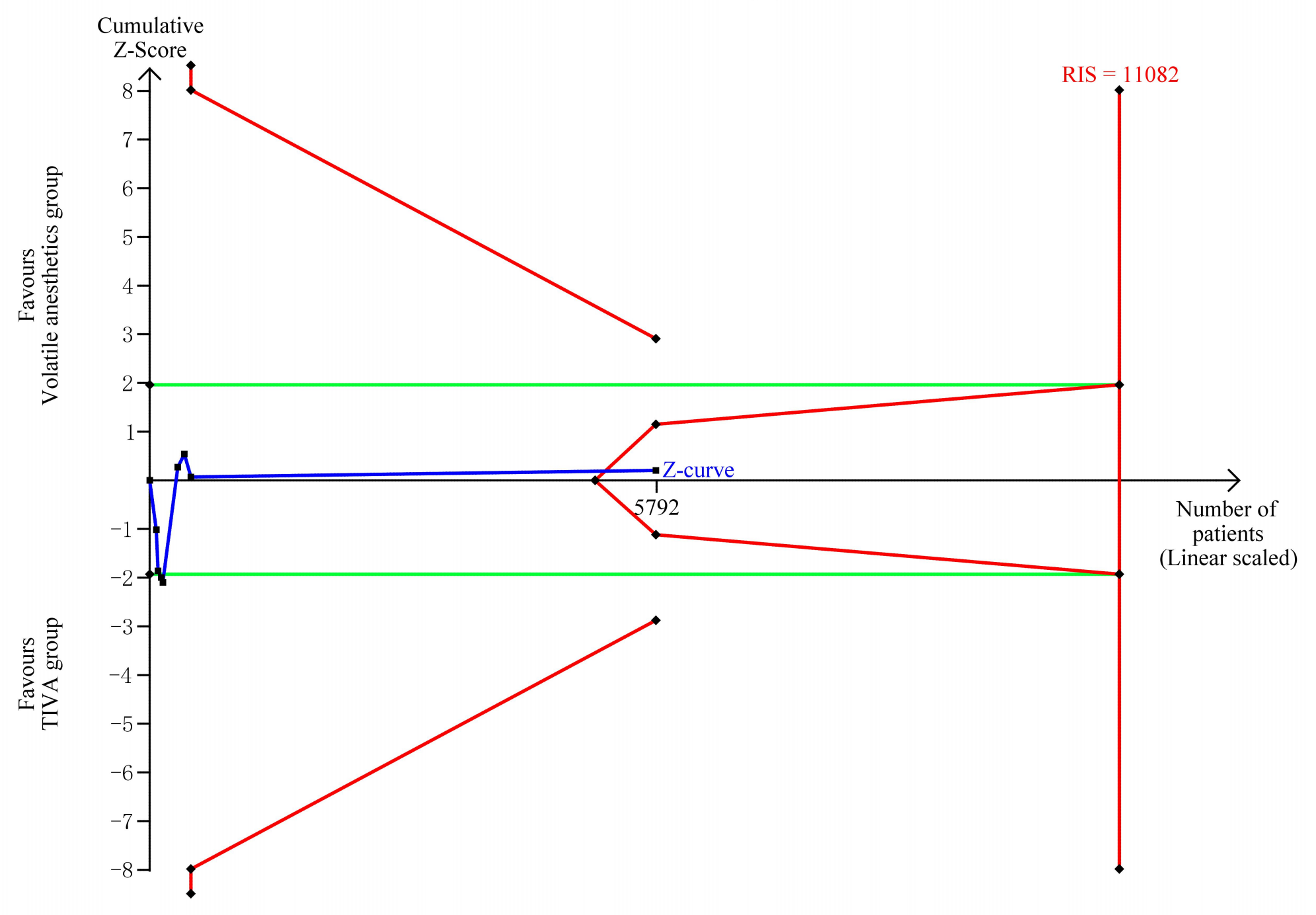

Supplement: S9 Fig — The risk of type Ⅰ error was set at 5% with a power of 80%. The variance was calculated from the data obtained from the included trials. The relative risk reduction (RRR) was set at 20%. (TIF) [file pone.0224562.s014.tif]

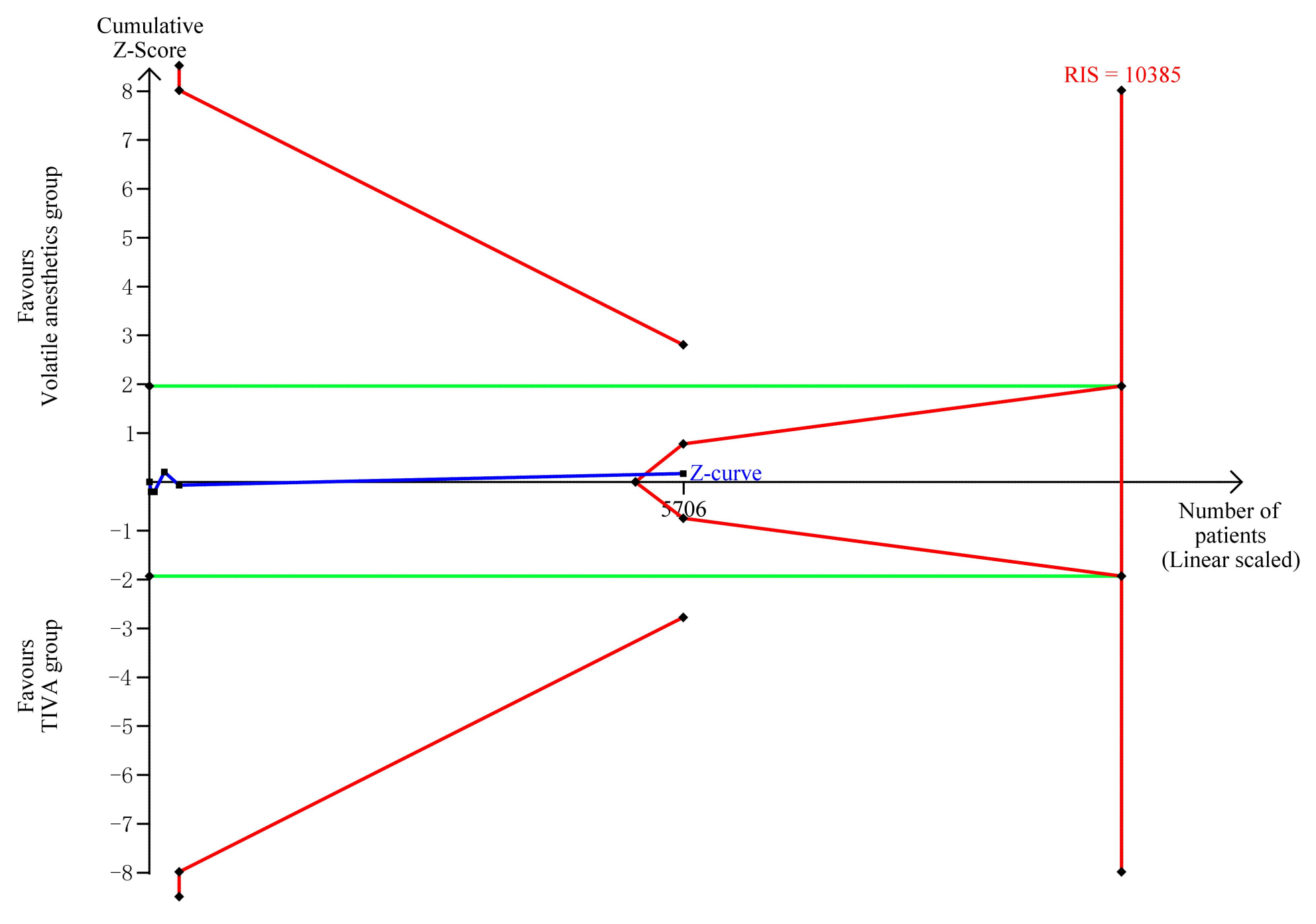

Supplement: S10 Fig — The risk of type Ⅰ error was set at 5% with a power of 80%. The variance was calculated from the data obtained from the included trials. The relative risk reduction (RRR) was set at 20%. (TIF) [file pone.0224562.s015.tif]

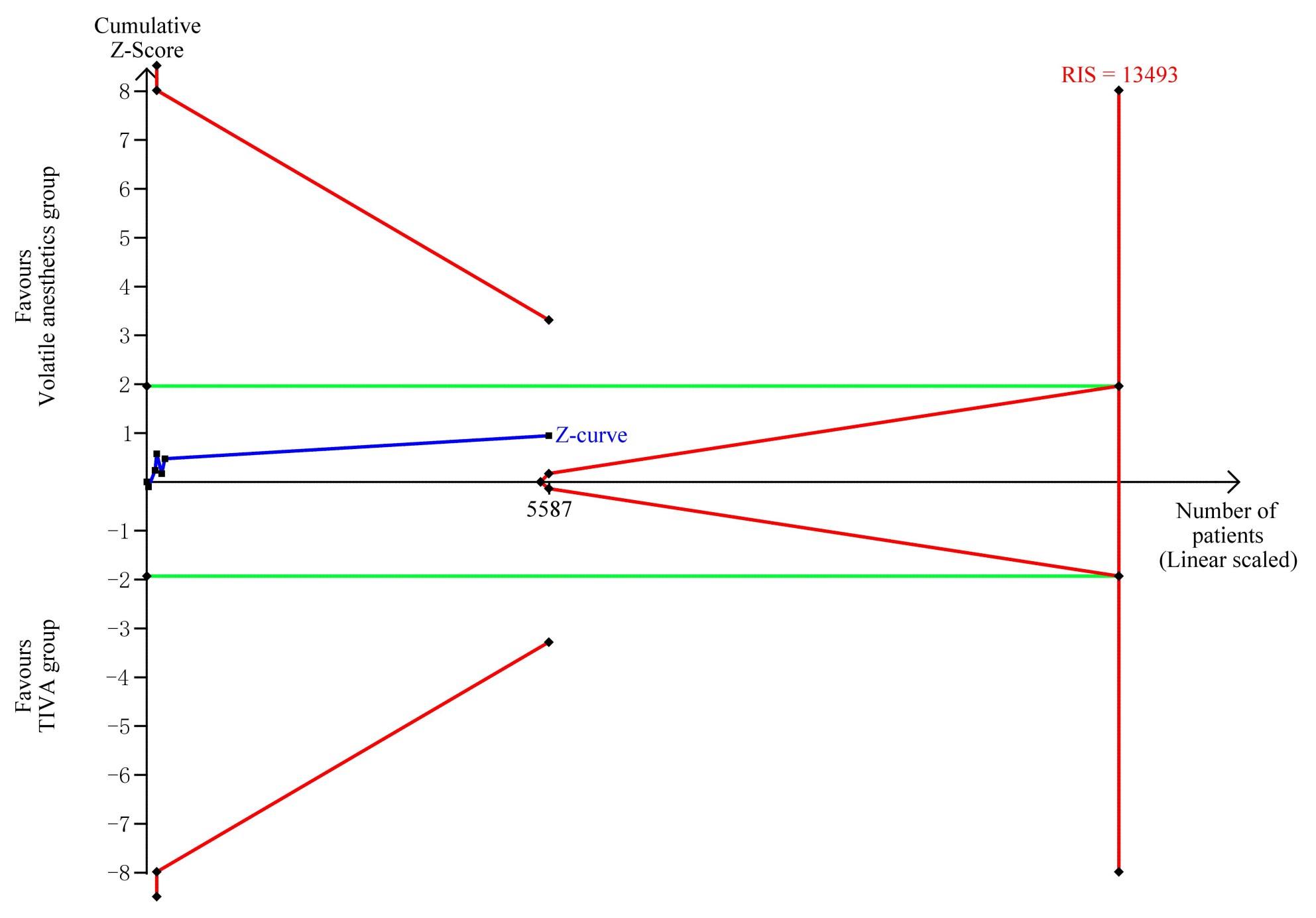

Supplement: S11 Fig — The risk of type Ⅰ error was set at 5% with a power of 80%. The variance was calculated from the data obtained from the included trials. The relative risk reduction (RRR) was set at 20%. (TIF) [file pone.0224562.s016.tif]

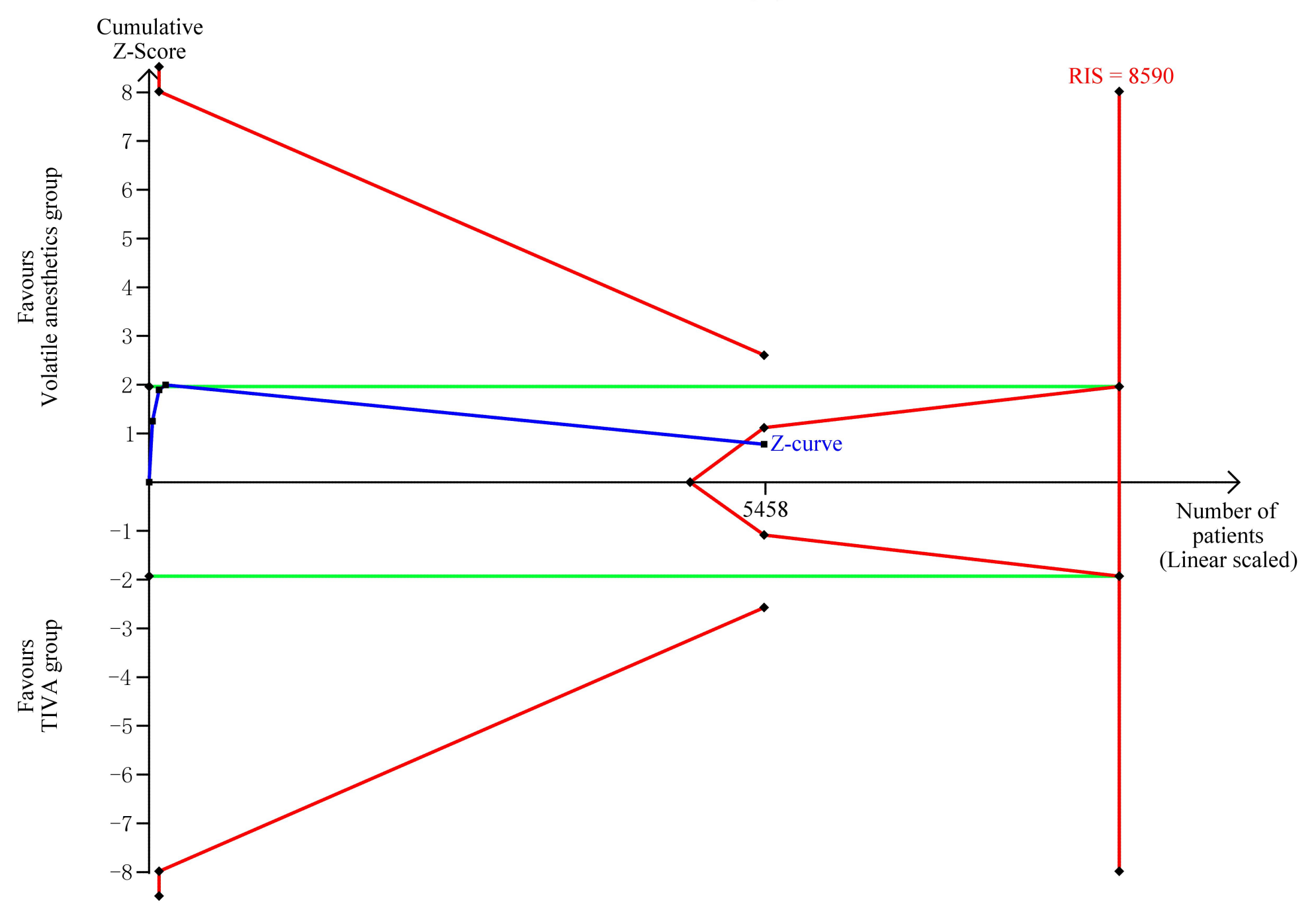

Supplement: S12 Fig — The risk of type Ⅰ error was set at 5% with a power of 80%. The variance was calculated from the data obtained from the included trials. The relative risk reduction (RRR) was set at 20%. (TIF) [file pone.0224562.s017.tif]
